# The New Molecules Are Changing the Course of Pediatric Chronically Active Ulcerative Colitis: A Series of Pediatric Cases

**DOI:** 10.1097/PG9.0000000000000100

**Published:** 2021-07-12

**Authors:** Rafael Martín-Masot, Pilar Ortiz Pérez, Encarnación Torcuato Rubio, Javier Blasco Alonso, Marta Herrador López, Carmen Gallego Fernández, Víctor Manuel Navas-López

**Affiliations:** From the * Pediatric Gastroenterology and Nutrition Unit, Hospital Regional Universitario de Málaga, Málaga, Spain; † Pharmacy Department, Hospital Regional Universitario de Málaga, Málaga, Spain.

**Keywords:** ulcerative colitis, small molecules, inflammatory bowel disease, colectomy

## Abstract

Chronically active ulcerative colitis (UC) constitutes a challenge in an era where medical therapeutic options have increased while experience with colectomies has decreased. The change in the therapeutic paradigm of the disease means that patients with chronically active UC are being managed waiting to find their therapeutic target. We present 2 cases of children with chronically active UC who did not respond to intravenous steroids nor sequential therapy. A response was obtained with ustekinumab and tofacitinib, 2 drugs widely used in adults but still with little evidence in children. Highlighting the important role of patients and their families helped decision-making, facilitating the work of the medical team. With multidisciplinary management and close follow-up, they have been able to avoid surgery entering complete clinical remission.

## Abstract 2

Supplemental Digital Content is available in the text.

**What Is Known?**

- In recent years, new therapeutic options for the treatment of pediatric inflammatory bowel disease have appeared.
- UStekinumab and tofacitinib may be valid options for the treatment of chronically active ulcerative colitis in children in the case of failure of anti-TNF treatment.
- The experience with these drugs is scarce in pediatric age.

**What Is New?**

- The treatment of the disease is changing, with a higher percentage of active chronic patients awaiting their therapeutic target.
- The work of a multidisciplinary team and the inclusion of the family in decision-making are essential for the management of active chronic patients.

## INTRODUCTION

Acute severe ulcerative colitis (ASUC) is a medical emergency defined as a Pediatric Ulcerative Colitis Activity Index (PUCAI) >65 points (1), being more frequently refractory to medical treatment in pediatric age (2). Up to 10% of patients have chronically active UC (3) and, although they have decreased, the rates of colectomy per year remain very high in ASUC, around 30% (4). Among patients who do not respond to intravenous corticosteroid therapy, some studies estimate colectomy rates as 50% in the first 2 years (5), and around 50%–60% of responders to the second-line therapy, calcineurin inhibitors or infliximab, will require colectomy within 1–2 years (5).

The therapeutic arsenal for the treatment of patients with UC has increased in recent years and, although most of the studies refer to the adult population, and the experience with the new molecules is also increasing in children (6).

Sequential therapy of calcineurin inhibitors after infliximab or vice versa might be considered in selected steroid-resistant ASUC patients in highly specialized centers. There have been no reports of sequential therapy in pediatric ASUC to date in the literature (7). In those patients with chronically active UC not responding to anti-TNF and vedolizumab, elective colectomy should be considered. The available evidence of the safety and efficacy of tofacitinib and ustekinumab in children with UC is scarce, and this is the main reason why the guidelines published by Turner et al did not include, as a possible strategies, neither of the these treatments that were ultimately effective in described patients (1,8).

We present 2 patients with chronically active UC, who responded to treatment with ustekinumab and tofacitinib. Golimumab, vedolizumab, and ustekinumab were used as “off-label” treatments, while tofacitinib required authorization as compassionate use, and informed consent was obtained from the parents before administration. Authors received permission from the parents of the patients in writing and they are aware of this article.

## CASE 1

**FIGURE 1.**
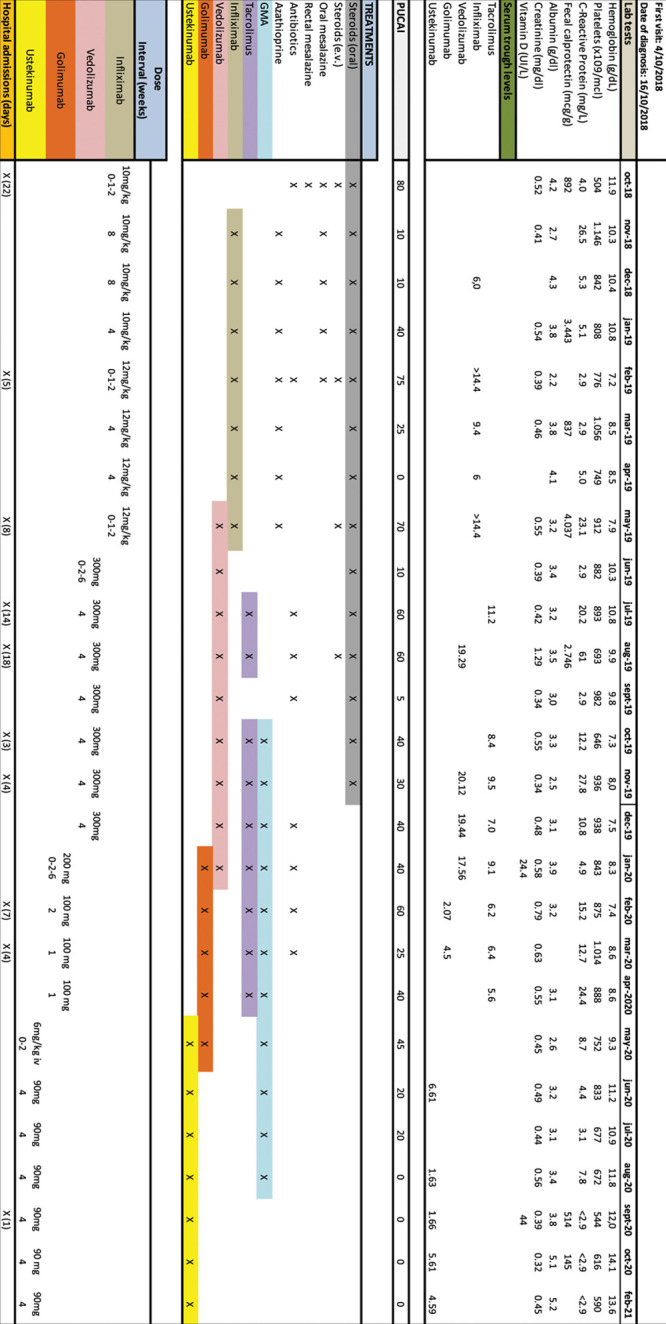
Patient 1 timeline including treatments, laboratory test results, PUCAI, and hospitalizations since diagnosis.

A 9-year-old girl with a 1-month history of abdominal pain, diarrhea, and weight loss. Laboratory tests were normal (Fig. 1) except fecal calprotectin (892 µg/g). Among the family history, a father with UC stands out. Colonoscopy showed macroscopic pancolonic involvement (Supplemental Digital Content 1, Figure 1A, B, http://links.lww.com/PG9/A54). Histology showed findings consistent with UC, and the PUCAI was 45 points. Oral prednisone was commenced along with oral and rectal mesalazine. The disease worsened over the following 10 days, she was admitted (PUCAI = 80 points) and intravenous corticosteroids were started. Five days later, infliximab 10 mg/kg/d with an accelerated 0-1-2 induction schedule was initiated. After 22 days of admission, after having received two transfusions of packed red blood cells, she was discharged (PUCAI = 10 points), receiving treatment with azathioprine, oral prednisone and IFX, 10 mg/kg every 4 weeks.

In the following 3 months, she required 2 additional hospital admissions, despite optimization of IFX (TL > 14.4 ng/mL] not being able to wean her off steroids. Infliximab was stopped and tacrolimus (0.1 mg/kg BID) and vedolizumab (VDZ) 300 mg intravenously with a 0-2-6 schedule were added. Trough plasma levels of tacrolimus were maintained between 5 and 12 ng/mL. One month later, she presented a severe flare, requiring a new hospital admission. Intravenous steroids were reinitiated, and antibiotic therapy (Jerusalem cocktail: vancomycin, amoxicillin, metronidazole, and doxycycline) (9) was also administered. The evolution was satisfactory, remaining hospitalized for 18 days. Two months later, she was still active despite treatment with steroids, tacrolimus and vedolizumab. Granulocyte-monocyte apheresis (GMA, Adacolumn), 1 session per week was started. After 8 sessions, oral steroids were weaned off but she was still moderately active with PUCAI of 40 points, requiring blood transfusions every 2 weeks. After 36 weeks of treatment with VDZ, the patient was switched to golimumab and the apheresis sessions were increased up to 2 weeks.

In the following 4 months, she required 2 hospital admissions due to moderate–severe flares. Due to lack of response after 6 months, golimumab was stopped and ustekinumab was started. A 6-mg/kg intravenous induction dose followed by 90 mg every 4 weeks subcutaneously as maintenance dose was prescribed. After 12 weeks, GMA could be stopped, when she was in clinical remission (PUCAI = 0 and C-reactive protein < 2.9 mg/L). The patient gained 8 kg of weight. Figure 1 shows different treatments used and the laboratory values of the patient in the last 2 years.

## CASE 2

**FIGURE 2.**
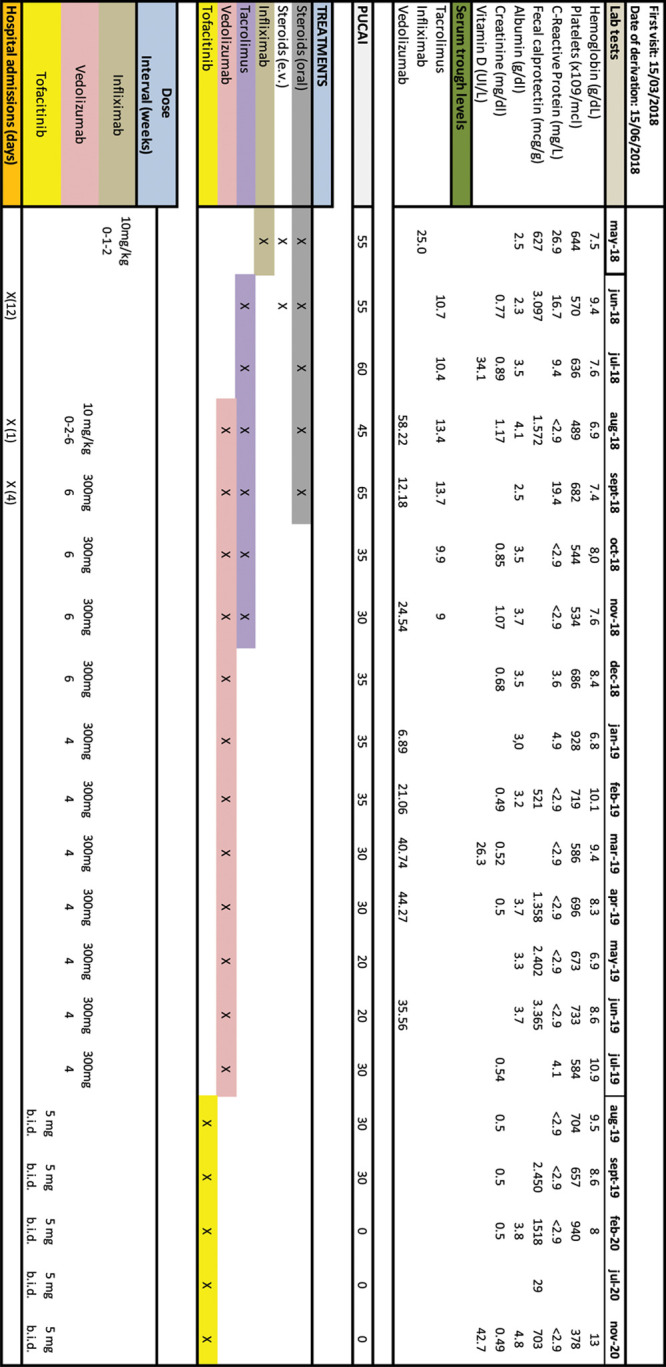
Patient 2 timeline including treatments, laboratory test results, PUCAI, and hospitalizations since diagnosis.

A 9-year-old boy with left-sided UC diagnosed based on typical endoscopy, histology, and elevated FC (Supplemental Digital Content 1, Figure 1C, D, http://links.lww.com/PG9/A54). Cytomegalovirus was suggested on biopsies by positive polymerase chain reaction and presence of inclusion bodies. Given moderately active disease at diagnosis (PUCAI = 40) treatment with prednisone, mesalazine, and ganciclovir was started. Disease worsened and he was admitted receiving treatment with intravenous corticosteroids. Given the lack of response in the following 5 days (PUCAI = 70), treatment with IFX (10 mg/kg, 0-1-2) was started. Despite adequate IFX trough levels (25 μg/mL), remission was not achieved and an antibiotic Jerusalem cocktail (vancomycin, amoxicillin, metronidazole, and doxycycline) (9) and tacrolimus (0.1 mg/kg BID) were added. In the following 14 days, a decrease of >20 points in the PUCAI was observed but clinical remission was not reached. In routine abdominal ultrasound, a hypoechoic nodule was detected in the spleen pole. Subsequently, a computed tomography of the abdomen and thorax showed nodular lesions in the lower lobe of the right lung, compatible with necrobiotic nodules (Supplemental Digital Content 2, Figure 2, http://links.lww.com/PG9/A55). VDZ 300 mg intravenously with a 0-2-6 scheme was started, without observing remission after 50 weeks of treatment, so it was withdrawn. After 6 months of treatment with VDZ, nodules were not visualized on ultrasound. During these 50 weeks, PUCAI was around 20–40 points, the tacrolimus dose was being adjusted, requiring withdrawal periods due to renal toxicity (creatinine 1.17 mg/dL), and blood transfusions were also needed. Tacrolimus and VDZ were stopped and tofacitinib 5 mg BID was started, with a progressive improvement achieving a complete clinical remission at week 20. After 16 months on tofacitinib (5 mg BID) treatment, the patient maintained clinical remission (PUCAI = 0 points). There has been no alteration of the lipid profile since the start of the drug. Figure 2 shows the different treatments used and the laboratory values of the patient in the last 2 years.

## DISCUSSION

We present 2 cases of patients with chronically active UC in whom colectomy was avoided with ustekinumab and tofacitinib.

There are currently several approved treatments for UC in children, such as adalimumab or infliximab. The use of drugs in pediatric IBD patients such as ustekinumab, vedolizumab, and golimumab are considered off-label, while tofacitinib requires compassionate use authorization for its use in children. The experience of ustekinumab and tofacitinib (10) use in UC in children is scarce. Dayan et al (11) reported data on the use of ustekinumab in 52 children with IBD of whom 4 (8%) with UC. It was shown as a safe option, obtaining better data in patients naive to anti-TNF. At week 52, 60% of patients with UC or unclassified IBD achieved clinical remission, and 50% were on steroid-free remission. Dhaliwal et al (12) treated 25 UC children with ustekinumab, all patients had failed prior infliximab therapy, and 12 also vedolizumab. At week 52, 11 out of 25 achieved clinical remission and 4 underwent colectomy. They also found that higher ustekinumab levels were not associated with a superior rate of clinical remission.

The dose used was according to the technical data sheet in induction by weight and 90 mg every 8 weeks subcutaneously. Unlike this study, in the first described patient, it was used at a dose of 90 mg every 4 weeks in maintenance. It was decided to establish ustekinumab in the presence of a central catheter for apheresis, which could increase the risk of thrombosis, being a fact to take into account in these patients before starting other drugs, such as tofacitinib, in addition to the good experience reported. Maintenance levels higher than 1.3 μg/mL have been associated with higher clinical remission rates in adults with UC (13).

Regarding the use of GMA, its use has been described in several series of patients, presenting variable response rates, between 50% and 80% (14). According to the guide for the management of UC from the Porto Group (15), its use could be considered in steroid-refractoriness or steroid-dependence cases. There is more experience in adult patients, although the results are also contradictory (16). In the first of 2 described cases, its use was beneficial, since corticosteroids could be withdrawn, although insufficient. On the other hand, although golimumab and vedolizumab have shown good results in patients with moderate and severe UC in pediatric age (17,18), they were not effective in the described patient.

In the second patient, it was decided to start tofacitinib, which has an easy dosage, and good outcomes have been described with its use, including in pediatric age. Dolinger et al reported that after a mean of 16 weeks of treatment with tofacitinib, 3 out of 5 patients were in steroid-clinical remission (19). This patient remains in steroid-free clinical remission, with no changes in the lipid profile, after >52 weeks of follow-up.

Colectomy rates have decreased in patients with UC, especially elective surgeries (20). Some studies of time trend have established a critical point in 2014 with the decline in colectomy rates, which could correspond with the advent of new molecules. In the cases that we present, without the availability of these drugs, both patients would have had to undergo surgery to treat their IBD.

An exact moment of when to go from medical to surgical treatment has not been established, except in emergency cases such as toxic megacolon or in cases of dysplasia. The decision, in a patient with chronically active UC, to terminate the medical treatment options and indicate elective surgery is not easy. This decision is conditioned mainly by 3 factors, the first of which is how long the treatment should be maintained until a response is obtained (21). A second factor is the experience of the multidisciplinary team, in close collaboration with the Hospital Pharmacy service. And last but not least, the family plays a central role in decision-making, and we believe that it must be made a participant in the process. Although studies on quality of life in adults show good results after colectomy (22), there is no experience in children. In the 2 cases that we present, the parents knew, at all times, the different therapeutic options, including surgery. Surgery in children with UC can have important consequences in terms of growth, but infertility has also been described as a sequel to surgery (23), and this is an aspect to consider, especially in girls. Furthermore, colectomy at this age presents a high rate of posterior pouchitis, and the results being related to the experience of the surgical center (24). On the other hand, medical treatment has some risks. The risk of immunosuppression and opportunistic infections must be taken into account, as well as an increased cardiovascular risk with the use of tofacitinib, as in the second patient.

A posteriori pharmacogenomic study was performed in both patients, observing favorable polymorphisms for long-term response with anti-TNF (25), which it did not correspond to what happened to described patients (Supplemental Digtal Content 3, Table 1, http://links.lww.com/PG9/A56). Although these pharmacogenetic data currently lack clinical applicability, their use in the future will probably be a cost-effective and hopeful strategy, since markers are necessary to predict the response to drugs, avoiding their prolonged use unnecessarily. In our opinion, the development and appearance on the market of new molecules is changing the management of pediatric IBD and severe UC. Except in life-threatening situations and if the appropriate circumstances are met (center with experience, multidisciplinary approach, and collaboration with the family), patients with chronically active UC are likely to benefit from conservative management, waiting to find their therapeutic target. Probably, the increased and better use of biologics and small molecules will change the profile of UC patients under follow-up, with professionals being responsible for the management of more chronically active patients instead of patients with the sequelae derived from undergoing surgery in pediatric age. We believe that it is essential to centralize the most complicated cases in experienced centers. Likewise, the decrease in colectomy rates will make it a challenge to unify the experience in reference surgical centers, to offer the best results in selected cases that require it. It is necessary to establish serological, immunological, or genetic biomarkers that predict the patient’s response to different therapeutic options, and that allow the determination of the patient’s therapeutic target and carry out a more proactive and personalized medicine. In conclusion, ustekinumab and tofacitinib may be valid options for the treatment of chronically active UC in children and should be considered in those patients who had failed before anti-TNF and vedolizumab therapy before considering colectomy.

## ACKNOWLEDGMENTS

All authors equally contributed to the design, preparation, and revision of the manuscript.

## Supplementary Material


