# Supplementary figures and images for: The New Molecules Are Changing the Course of Pediatric Chronically Active Ulcerative Colitis: A Series of Pediatric Cases
Source: JPGN Rep. 2021 Jul 12;2(3):e100. doi: 10.1097/PG9.0000000000000100 (PMC10191510; doi:10.1097/PG9.0000000000000100)

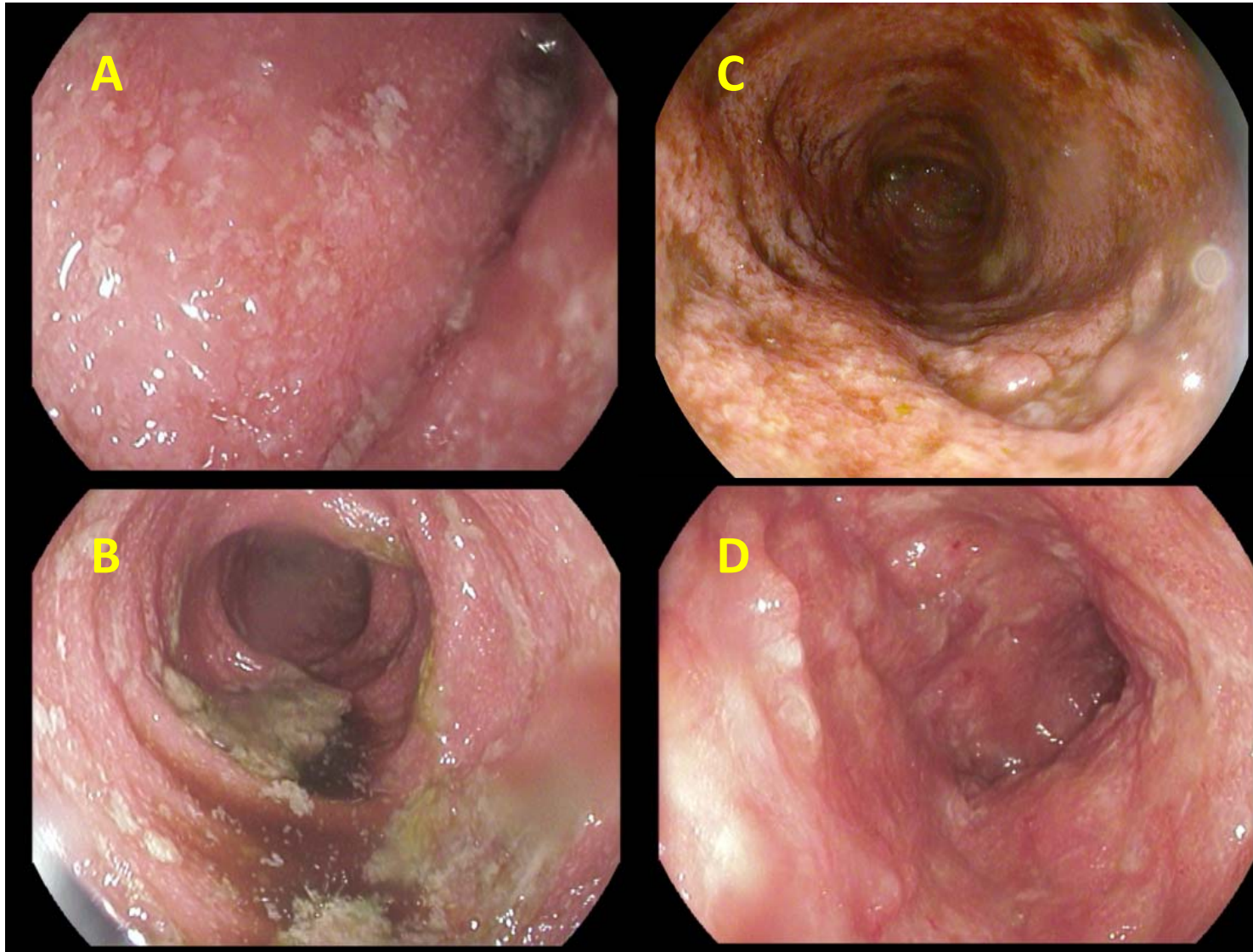

Supplement: Supplementary file 1 [file pg9-2-e100-s001.pdf]

**A**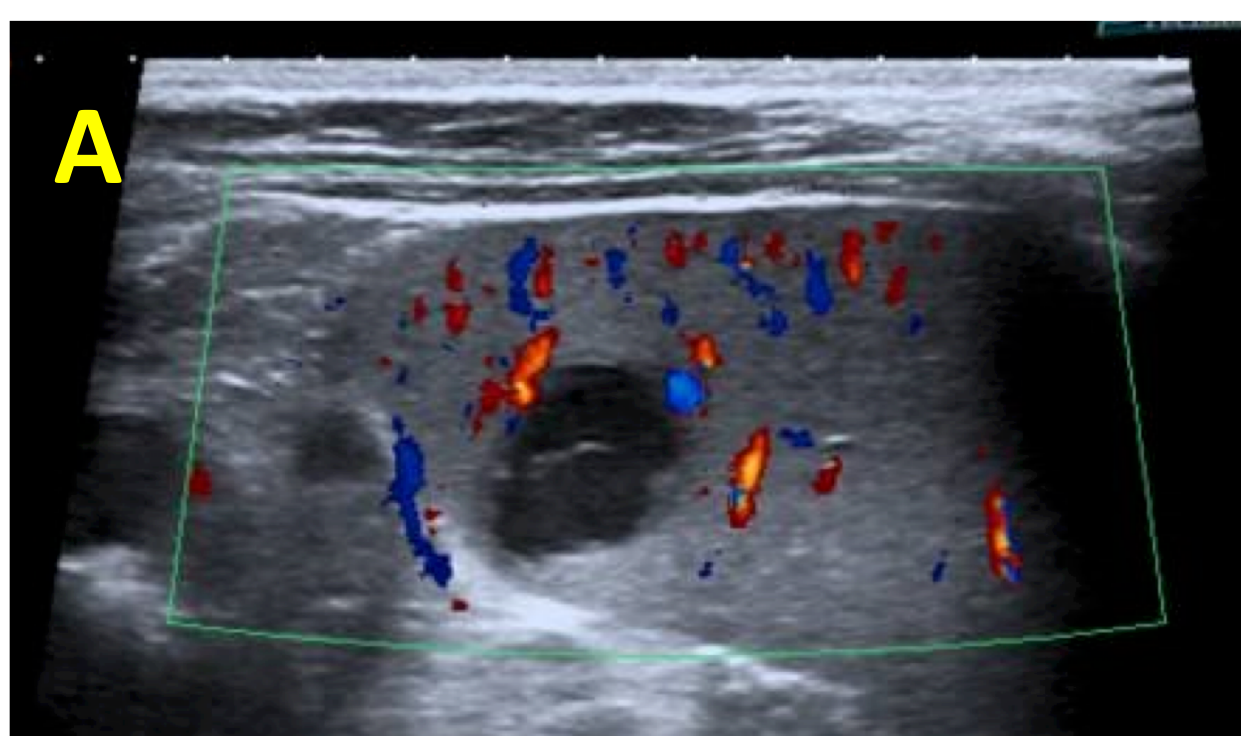**B**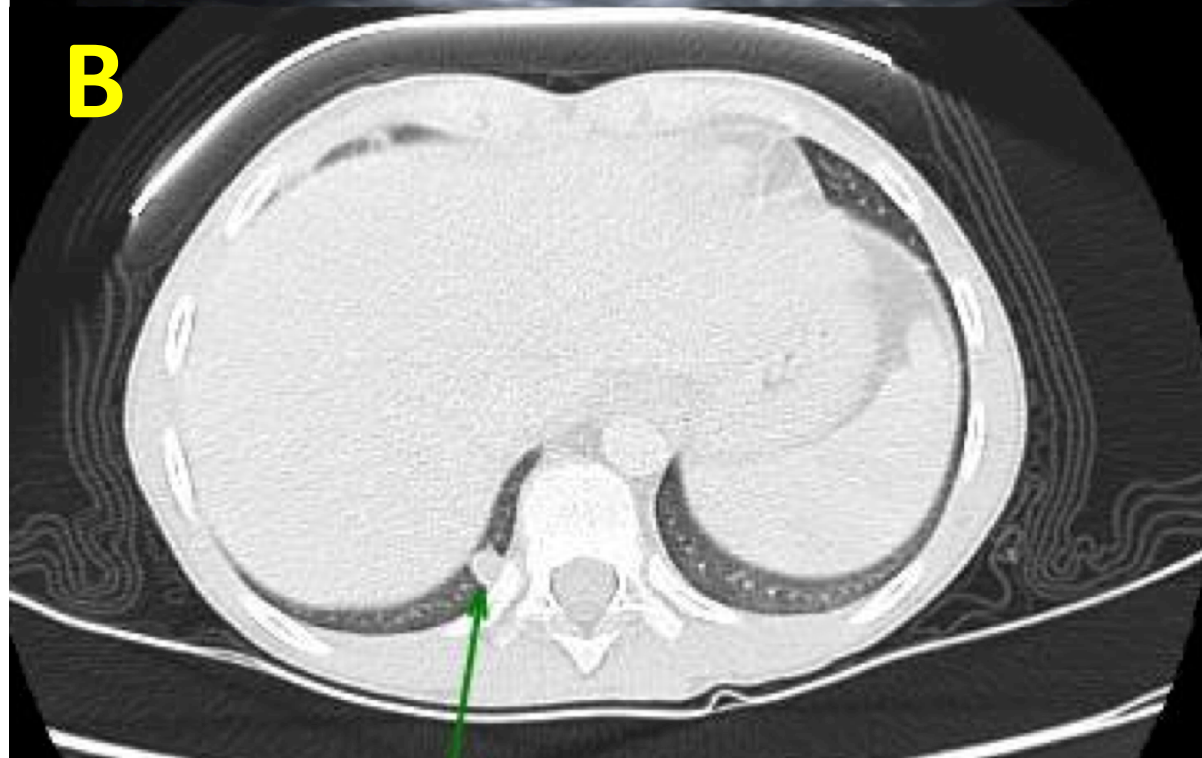**C**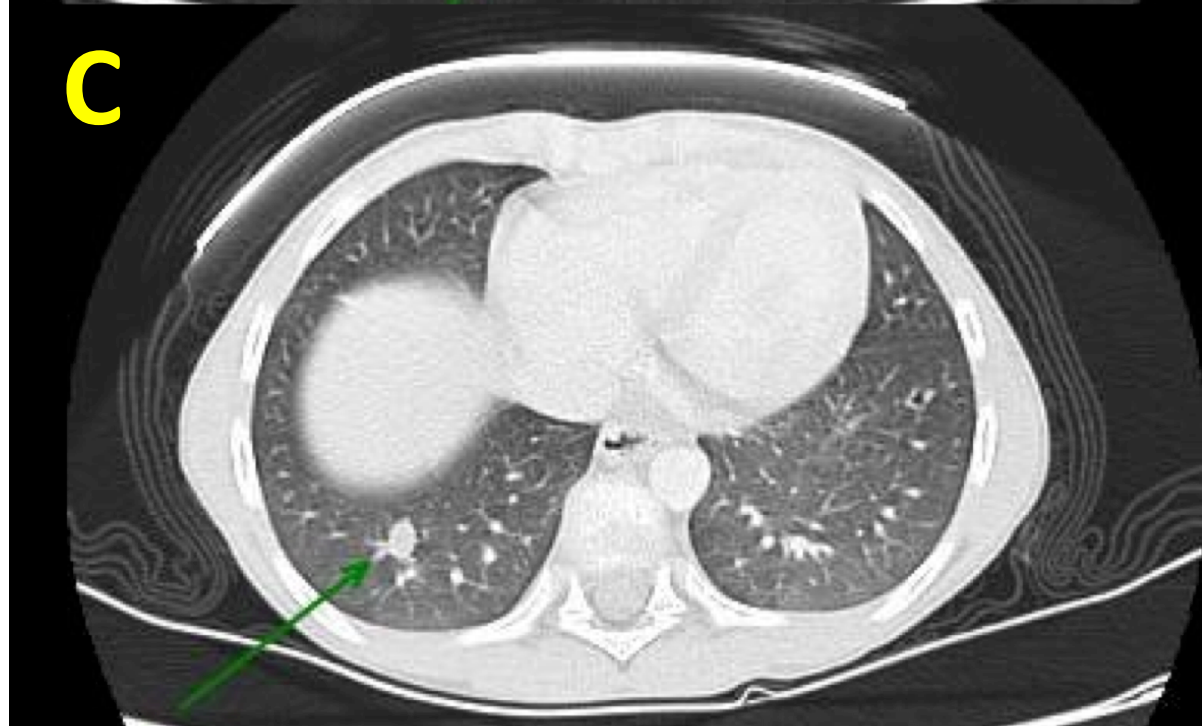

Supplement: Supplementary file 2 [file pg9-2-e100-s002.pdf]
