# Supplementary material for: The New Molecules Are Changing the Course of Pediatric Chronically Active Ulcerative Colitis: A Series of Pediatric Cases
Source: JPGN Rep. 2021 Jul 12;2(3):e100. doi: 10.1097/PG9.0000000000000100 (PMC10191510; doi:10.1097/PG9.0000000000000100)
Supplement: Supplementary file 3 [file pg9-2-e100-s003.pdf]

Supplementary table 1: DNA sequence variations in genes that may be involved in the response to infliximab.

Gen (analyzed variants)	Patient 1	Patient 2
**_TLR2_ rs1816702**	CC	CC
**_TLR2_ rs3804099**	CT	CT
**_TLR4_ rs5030728**	AG	GG
**_TLR9_ rs352139**	AG	AG
**_LY96_ rs11465996**	CC	CC
**_IL10_ rs1800872**	CC	CA
**_IL10_ rs3024505**	CT	CC
**_IL6_ rs10499563**	TC	TT
**_TNFRSF1A_ rs4149570**	GT	TT
**_TNFAIP3_ rs6927172**	CG	CC
**_CD14_ rs2569190**	AG	Not performed
**_MAP3K14_ rs7222094**	TT	Not performed
**_TNFA_ rs361525**	GG	GG
**_IL1B_ rs4848306**	GA	GG
**_IL17A_ rs2275913**	GG	AA
**_TNF1B_ rs1061622**	TT	TG
**_TNF1B_ rs1061624**	GG	GG
**_TNF1A_ rs767455**	CT	TT
**_TNF_ rs1800629**	GG	GG
**_FASL_ rs762000**	CT	Not performed
**_FCGR3A_ rs396991**	Indeterminate	Not performed
**_TNF1B_ rs3397**	TT	TT

Gen (analyzed variants)	Genotype		Interpretation
**_LY96_ (rs11465996)**	CC/CG		Greater probability of long-term response to anti-TNFs
**_TLR4_ (rs5030728)**	GG	40.7%	Probability of having infliximab levels below 3 µg / mL
	GA/AA	17%	
**_IL10 (rs3024505)_**	CC	19%	
	CG/GG	46.5%	
